# Reply to “Marine abundance and its prehistoric past in the Baltic”

**DOI:** 10.1038/s41467-022-30151-8

**Published:** 2022-05-20

**Authors:** J. P. Lewis, D. B. Ryves, P. Rasmussen, J. Olsen, L. G. van der Sluis, P. J. Reimer, K.-L. Knudsen, S. McGowan, N. J. Anderson, S. Juggins

**Affiliations:** 1 Geography and Environment, Loughborough University, Loughborough, UK; 2 Environmental Archaeology and Materials Science, National Museum of Denmark, Brede Værk, Lyngby, Denmark; 3 Department of Physics and Astronomy, Aarhus University, Aarhus C, Denmark; 4 School of Natural and Built Environment, Queen’s University Belfast, Belfast, UK; 5 Department of Earth Science, Aarhus University, Aarhus C, Denmark; 6 School of Geography, University of Nottingham, Nottingham, UK; 7 Department of Aquatic Ecology, Netherland Institute of Ecology (NIOO-KNAW), Wageningen, Netherlands; 8 School of Geography, Politics and Sociology, Newcastle University, Newcastle upon Tyne, UK

**Keywords:** Palaeoecology, Palaeoclimate

**replying to** Hausmann et al. *Nature Communications* 10.1038/s41467-022-30150-9 (2022)

In response to the comment by Hausmann et al.^1^ we highlight here that a number of the key criticisms of Lewis et al.^2^ are either misinterpretations of our paper or are speculative, requiring rigorous testing via empirical data (and subsequently are topics for further research). We would, therefore, like to take the opportunity to clarify these points, so that others do not misinterpret our study^2^ in the same way. Hausmann et al.^1^ provide no physical evidence or data that rebuke our hypothesis, and therefore in the spirit of critical scientific discussion and endeavour, we challenge them (or others) to disprove our hypothesis through high-quality data, and hope that our original paper^2^ and this further discussion stimulate such work. The criticisms expressed by Hausmann et al. largely focus on the use of a summed probability distribution ^14^C curve based on oysters as a proxy for shell midden abundance, yet this is only a supportive dataset within the broader theme of this study, and we certainly welcome future research into improving how we quantify shell midden abundance and marine resource intensification in past cultures and societies. However, we highlight that the criticisms of this ^14^C oyster-derived dataset by Hausmann et al.^1^ does not detract from the key point of this study, that population increased during periods of increased marine productivity (demonstrated by sediment pigment and other proxy data) and hence increased marine resource availability, when humans predominately consumed a marine-based diet^3^. Below we respond to the specific points raised by Hausmann et al.^1^.

## Shell accumulations and radiocarbon dates as proxy measures of marine consumption

Hausmann et al.^1^ claim that the method of using shell middens as a proxy measure of marine consumption does not work for Southern Scandinavia due to variable size/volumes of shell deposits and uncertain taphonomy, but do not present any data or evidence from Southern Scandinavia to support this claim. The assertions by Hausmann et al.^1^ are based on local variation in a few targeted-accumulation rate (AR) studies in locations far from our study sites. We are, of course, aware of the complexities that result from taphonomic processes and that local variability in shell midden ARs will occur across Southern Scandinavia. Furthermore, our article does not state that there are ‘no reliable methods available’ for measuring ARs in a shell midden, (e.g. Stein et al.^4^ highlighted by Hausmann et al.^1^), and we acknowledge that such studies^3^, if applied widely, offer potential for improving our understanding of human-resource exploitation over the study area. However, as these types of detailed quantitative data are unavailable for most shell middens/deposits across Southern Scandinavia, other methods for estimating this broader-scale temporal abundance are needed. Our geographically widely-distributed ^14^C dataset, based on data generated over half a century through the work of various researchers, can only be fully rejected with the collection of relevant data that refute higher shell midden abundance during the two such phases we identify as P1 (7600–7100 cal. yr BP) and P2 (6400–5900 cal. yr BP)^2^, and/or that there were higher levels of shell midden destruction in the preceding or succeeding periods. Our data are in good agreement with archaeological observations of trends over the study period^5^. The ^14^C-based shell midden abundance curve^2^ (used as a proxy for the intensity of marine resource utilisation), shows clear correlation with the other datasets presented^2^, including human population increase that coincides with an increase in marine productivity, at a time where isotope evidence suggests a predominantly marine diet^3^. When marine primary production is high^2^, biota and biomass at higher trophic levels will almost certainly increase^6^ and therefore marine resources will generally be more abundant. This will provide a wide and richer resource base, necessary for human demographic growth.

## Sea-level variation and site preservation

**Fig. 1:**
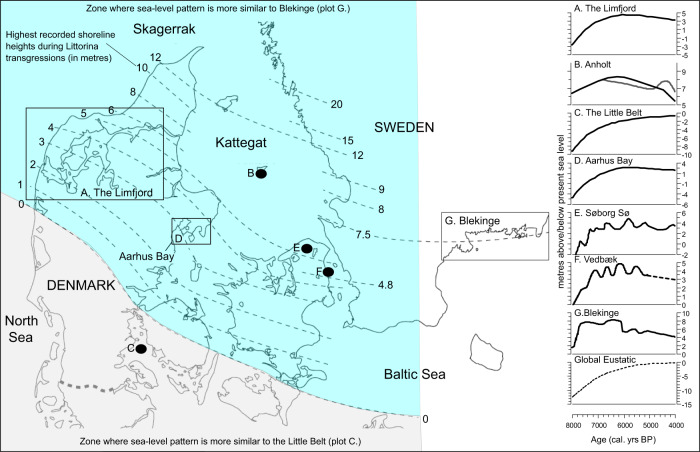
Sea-level history of Southern Scandinavia. Map showing highest shoreline height during Littorina transgressions^8^, various sea-level curves^7,9,20,23–27^ from Southern Scandinavia over the study period (8000–4000 cal. yr BP) and global eustatic sea-level change^28^. Note the variability in sea-level curves in terms of transgressive and regressive phases and sea-level maxima.

Hausmann et al.^1^ claim that the Swedish Blekinge sea-level curve is only ‘broadly representative’ (quoting Lewis et al.^2^) for ‘northern Denmark’, and that ‘the southern half of the country’ has a different sea-level curve more like the Little Belt sea-level curve^7^ as a comparison (Fig. 1 in Hausmann et al.^1^). In Fig. 1, we show the shoreline heights (based on Bjørnsen et al.^8^) and multiple sea-level curves for the study period. Above the 0 m line in Fig. 1 (shaded in blue), sea-level curves follow a pattern more like that from Blekinge (Berglund et al.^9^) making up the majority (and certainly far more than ‘half’) of Southern Scandinavia. Below this line, land subsidence means the Little Belt-type sea-level curve^7^ is more relevant, thus limiting the area available for discovery of in situ middens and other coastal/marine sites. Submerged archaeology is still not performed on a larger scale, with most discoveries to date being single finds that are often disturbed or re-deposited^10^. To our knowledge, the few submerged shell middens analysed from this region do not contradict our findings and no such evidence is presented in Hausmann et al.^1^. We naturally agree that submerged archaeology is an interesting and rapidly developing field that will likely yield some important, additional data on human habitation and resource exploitation during the Holocene in Southern Scandinavia, and we welcome Hausmann et al.’s call to expand and extend such studies.

**Fig. 2:**
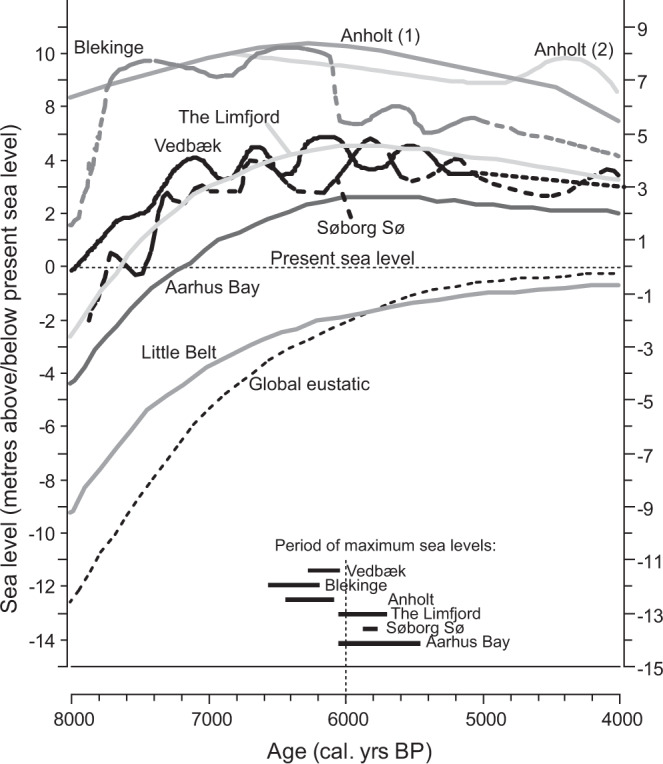
Summary of available sea-level curves for Southern Scandinavia. Sea-level curves for Southern Scandinavia^7,9,20,23–27^ are plotted on common altitude scale, with period of maximum sea-levels highlighted to show variability.

We agree that, across the region, maximum sea levels were likely responsible for the destruction of some middens from earlier periods and that this needs to be tested more thoroughly. However, between 7500 and 4000 cal. yr BP (the focus of our study period) sea-levels are extremely variable across Southern Scandinavia^11^, due to changing local isostatic rates and localised eustacy. This results in geographically variable transgressive and regressive phases and sea-level maxima occurring anytime between ca. 6500 and 5500 BP (Figs. 1, 2 and references therein) and therefore sea-level driven taphonomy alone cannot explain the patterns in the oyster shell ^14^C dates curve. Therefore, with this sea-level variability in mind, perhaps in future research linkages between sea-level and shell midden abundance needs to be tested at a more local scale in areas where high-quality sea-level curves exist, to evaluate if the beginning of the rapid shell midden increase frequently post-dates the sea-level high-stand.

## Oyster shells as a proxy for resource availability and the wider marine economy

This criticism in Hausmann et al.^1^ misinterprets our hypothesis. It is widely accepted that the oyster was no more than a supplement, or seasonal resource^12^ (stated in Lewis et al.^2^) in the diet of the late Mesolithic Kongemose and Ertebølle cultures. We do not suggest that increased oyster availability and consumption infers marine resource intensification. Instead, we are inferring that both increased shell midden abundance (with the middens themselves containing a wide variety of marine resources) based on ^14^C dates on oyster shells, and isotope data showing a predominately marine resource based diet, imply increased usage of the abundant marine resources to support an increasing human population. We also acknowledge that, in addition to marine productivity and marine resource availability, other factors may also influence the degree of marine resource exploitation and the predominant resource(s) exploited (e.g. shellfish, marine mammals, birds fish), with local to regional variation.

Hausmann et al.^1^ state that our second assumption is that oysters are a good predictor of other marine resources. In fact, we argue that the high level of primary production (based on sedimentary pigment data^2^) is a good indicator of widely available marine resources. Hausmann et al.^1^ focus too strongly on oysters, and do not acknowledge that greater primary productivity will support increased production at higher trophic levels^6^. We, of course, agree that there are many other types of archaeological sites and natural archives which can provide important information on marine resource availability in addition to shell middens, but more data are needed on their number, age span, location, size and faunal content.

Concerning the recommendations of applying growth rates and size and mortality profiles of oyster shells and fish remains, or compound specific isotope analysis of amino acids, Hausmann et al.^1^ highlight themselves that few such analyses exist, certainly too few to be conclusive. For example, in the oyster-based age and size mortality studies cited by Hausmann et al.^1^, of the three shell middens analysed^13^, only Norsminde Fjord shows a clear decline in size and age of oysters beginning in the Neolithic period. The Krabbesholm shell midden only has data from one layer within the Mesolithic period for comparison, and the Havnø shell midden exhibits a decreasing trend in age and size of oysters beginning in the Mesolithic period (and with only data for 3 shell midden layers in total across the Mesolithic and Neolithic periods). We encourage that more such studies should be carried out to examine this aspect critically. Furthermore, as shellfish were only a supplement to the diet, then oyster populations may not have become visibly stressed until later (i.e. exhibiting changes in age/size), following demographic growth during the Neolithic boom period^14^, and when changing environmental conditions imposed greater physiological pressure on oyster populations^15^. We refrained from discussing the fate of marine resources in the Neolithic period, due to the added complexity associated with the introduction of agriculture, which was beyond the scope, and not the focus of our paper^2^.

## Biomolecular evidence of palaeodiet

Our article does not state that the people of the Maglemose culture did not fish, only that marine resources were of less importance to this cultural group in Southern Scandinavia. A further point of clarification may be needed here, in that we include freshwater resources within our grouping of terrestrial resources, as these are obtained from lakes and rivers within the terrestrial environment. Aquatic resources (particularly freshwater fish^16^) were clearly important within the Maglemosian diet, along with terrestrial plants and game^17^. We of course agree that Maglemosian groups at the coastal fringes most likely used marine resources and relevant fishing technology^18^ (as highlighted by Hausmann et al.^1^). But this does not undermine the key point about the relative importance of terrestrial and freshwater resources compared to marine resources for Maglemosian people. Given the geographical distribution of this culture, inhabiting a vast terrestrial landscape (encompassing the land bridge with Sweden) replete with abundant freshwater habitats, they would have largely used terrestrial and freshwater resources^16^, and relied much less on marine resources, except at the coastal fringes in the more northerly areas^18^. To the south, along the palaeo-shorelines of the western Baltic proper, these resources would also have been limnic to brackish up until ca. 8000–7700 BP^7,19–21^, prior to a permanent marine connection being established. When sea-levels rose and gradually flooded the wider landscape (9000–7600 cal. yr BP), turning Denmark into an archipelagic seascape (with a much greater coastal area), then there is clear evidence for the emergence of two cultures (first the Kongemose and later the Ertebølle culture) who predominately exploited marine resources^3^.

## Fishing technology

We thank Hausmann et al.^1^ for highlighting that older dates are available for some of the fishing technologies. However, we discuss technological advancements very tentatively within the paper and with reference to the ongoing, and very far from settled, debate concerning the complexity of Scandinavian late Mesolithic societies^22^. We agree that our links to technological advancement are preliminary, and that this complicated topic is in need of further research.
